# Efficacy and safety of piperacillin–tazobactam compared with meropenem in treating complicated urinary tract infections including acute pyelonephritis due to extended-spectrum β-lactamase-producing *Enterobacteriaceae*

**DOI:** 10.3389/fcimb.2023.1093842

**Published:** 2023-05-03

**Authors:** Wei Zhang, Chun-Yu Yan, Shu-Rui Li, Ting-Ting Fan, Shan-Shan Cao, Bin Cui, Meng-Ying Li, Bo-Yuan Fan, Bo Ji, Li Wang, Fei Cui, Jia Cui, Lei Wang, Yue Guan, Jing-Wen Wang

**Affiliations:** ^1^ Department of Pharmacy, Xijing Hospital, The Fourth Military Medical University, Xi’an, China; ^2^ Department of Metabolism, Digestion and Reproduction, Faculty of Medicine, Imperial College London, London, United Kingdom; ^3^ Department of Traditional Chinese Medicine, Tianjin University of Traditional Chinese Medicine, Tianjin, China; ^4^ School of Basic Medicine and Clinical Pharmacy, China Pharmaceutical University, Nanjing, China; ^5^ Department of Endocrinology, Xijing Hospital, The Fourth Military Medical University, Xi’an, China; ^6^ Department of Cardiology, The Second Affiliated Hospital of Xi’an Jiaotong University, Xi’an, China; ^7^ Department of Pharmacy, Yan’an University Affiliated Hospital, Yan’an, China; ^8^ Department of Pharmacy, Luoyang First People’s Hospital, Luoyang, China

**Keywords:** complicated urinary tract infections, piperacillin-tazobactam, ESBL, antibiotic treatment, meropenem

## Abstract

**Introduction:**

Extended-spectrum β-lactamase (ESBL)-producing Enterobacteriaceae pose a huge threat to human health, especially in the context of complicated urinary tract infections (cUTIs). Carbapenems and piperacillin–tazobactam (PTZ) are two antimicrobial agents commonly used to treat cUTIs.

**Methods:**

A monocentric retrospective cohort study focused on the treatment of cUTIs in adults was conducted from January 2019 to November 2021. Patients with a positive urine culture strain yielding ≥ 103 colony-forming units per milliliter (CFU/mL), and sensitive to PTZ and carbapenems, were included. The primary endpoint was clinical success after antibiotic therapy. The secondary endpoint included rehospitalization and 90-day recurrence of cUTIs caused by ESBL-producing Enterobacteriaceae.

**Results:**

Of the 195 patients included in this study, 110 were treated with PTZ while 85 were administered meropenem. The rate of clinical cure was similar between the PTZ and meropenem groups (80% vs. 78.8%, p = 0.84). However, the PTZ group had a lower duration of total antibiotic use (6 vs. 9; p < 0.01), lower duration of effective antibiotic therapy (6 vs. 8; p < 0.01), and lower duration of hospitalization (16 vs. 22; p < 0.01).

**Discussion:**

In terms of adverse events, the safety of PTZ was higher than that of meropenem in the treatment of cUTIs.

## 1 Introduction

Complicated urinary tract infections (cUTIs) are defined by the presence of systemic symptoms or by the susceptibility of the host to a complicated disease course (Geerlings et al., 2013). Systemic symptoms include fever, febrile UTI, and other symptoms indicative of tissue infections like pyelonephritis, prostatitis, or urosepsis syndrome (Geerlings et al., 2013). cUTIs occur in patients having a structural or functional abnormality in their genitourinary tract or through infection of urinary catheters (Melekos and Naber, 2000; Kongnakorn et al., 2019). Most of the challenging cUTIs are caused by Gram-negative bacteria, such as *Escherichia coli* and *Klebsiella pneumoniae*, that can resist multiple antimicrobial agents by producing extended-spectrum β-lactamase (ESBL) enzymes, such as CTX-M enzymes, AmpC β-lactamases, and carbapenemases (Chatterjee et al., 2021; Syed, 2021; Jean et al., 2022; López Montesinos et al., 2022; Wald-Dickler et al., 2022; Zilberberg et al., 2022). By prolonging hospitalization, cUTIs have imposed a serious burden on healthcare systems (Food and Drug Administration, 2018; Vallejo-Torres et al., 2018; Zilberberg et al., 2021).

The treatment options for cUTIs are distinct from those for uncomplicated UTIs. For decades, carbapenems—members of the broad-spectrum β-lactam family—have been identified as the gold standard antibiotic therapy for cUTIs caused by ESBL-producing *Enterobacteriaceae*. They potently inhibit cell wall biosynthesis by preventing transpeptidation in many Gram-positive, Gram-negative, and anaerobic bacteria (Zhanel et al., 2007; Bush and Bradford, 2016; Armstrong et al., 2021). Meropenem is an antibacterial agent from the carbapenem family that is commonly used in empirical therapy for serious infections before the causative organism has been identified (Wiseman et al., 1995; Baldwin et al., 2008).

Piperacillin is a β-lactam antibiotic that is commonly used to boost the antibacterial activity of tazobactam (Drawz and Bonomo, 2010). ESBL-producing *Enterobacteriaceae* are susceptible to β-lactam/β-lactamase inhibitor combinations, such as piperacillin–tazobactam (PTZ), which can inhibit ESBL enzymes that confer antibiotic resistance.

Meropenem and PTZ constitute a considerable part of the therapeutic regimens employed in clinical practice at Xijing Hospital. The real-world setting for the treatment of cUTIs involves complex medical complications and variable physical conditions, which make the choice of the optimum antibiotic regimen a challenging one. Retrospective studies with real-world data can help clinical practitioners deliver precise prescriptions in the future.

This study aimed to utilize real-world data assessing the efficacy and safety of β-lactam antibiotics (PTZ) versus carbapenems (meropenem) for the effective treatment of cUTIs caused by ESBL-producing *Enterobacteriaceae*.

## 2 Methods

### 2.1 Objectives

A monocentric and retrospective cohort study was conducted involving patients treated for cUTIs between January 2019 and November 2021. The objective of this study was to compare the efficacy and safety of PTZ and meropenem for treating cUTIs caused by ESBL-producing *Enterobacteriaceae*.

### 2.2 Study design

Adult patients were eligible if they were diagnosed with cUTIs caused by ESBL-producing *Enterobacteriaceae* that were non-susceptible to ceftriaxone or cefotaxime, but susceptible to PTZ and meropenem. Some patients may suffer from acute cystitis and acute pyelonephritis due to the administered interventions. Patients were treated with PTZ or meropenem for more than 72 h. General practitioners adapted the antibiotic dosage, administration route, and treatment duration for each individual based on their renal function as measured by their creatinine clearance rate.

### 2.3 Inclusion/exclusion criteria

The local research team screened patients manually using inclusion and exclusion criteria, and data were selected from patient medical charts using standardized case report forms.

#### 2.3.1 Inclusion criteria

Patients who met all the following criteria were included in this study:

- Age ≥ 18 years
- Documented cUTIs, with a positive urine culture strain ≥ 10^3^ colony-forming units per milliliter (CFU/ml) that is sensitive to PTZ and carbapenems. The cUTIs must be accompanied by at least two of the following signs and symptoms (Food and Drug Administration, 2018):
- chills or fever (temperature > 38°C)
- nausea or vomiting
- flank or pelvic pain
- dysuria, urinary frequency, or urgency
- costovertebral angle tenderness on physical examination (Delory et al., 2021)

#### 2.3.2 Exclusion criteria

Patients who met any of the following criteria were excluded from this study:

- Antibiotic treatment for less than 72 h
- Urine culture positive for Gram-positive bacteria at a colony count ≥ 10^5^ CFU/ml
- Urine culture positive for fungi at a colony count > 10^3^ CFU/ml
- Repeated exposure to the drugs used in this study

### 2.4 Study procedures

The experimental procedures included baseline urine collection for quantitative culture. The signs and symptoms of cUTIs and adverse events were routinely monitored. Prespecified laboratory data, including chemistry panels and urine cultures, were collected.

Baseline urine cultures at a colony count ≥ 10^5^ CFU/ml were sent to the central laboratory (Department of Laboratory Medicine, Institute of Clinical Laboratory Medicine of PLA, Xijing Hospital, Air Force Military Medical University) for identification, quantification, susceptibility testing, and further characterization of the organism(s). The minimum inhibitory concentrations (MICs) of PTZ and meropenem were determined using the agar dilution reference method, following the laboratory procedures and MIC breakpoints provided by CLSI (2021).

### 2.5 Study endpoints

The primary efficacy endpoint of this study was the clinical and microbiological response in patients with cUTIs at the end of the initial antibiotic regimens. The clinical response was defined by the Food and Drug Administration (FDA) as follows:

- Resolution of all core cUTI symptoms, including fever (temperature > 38°C), dysuria, urinary frequency, urinary urgency, suprapubic pain, and flank pain
- No appearance of new cUTI symptoms
- No further use of antibiotics with microbiological cure (urine culture at a colony count < 10^3^ CFU/ml, negative blood culture)

According to the FDA guidelines, the microbiological response was defined as a colony count of the bacterial pathogen, identified at trail entry, falling below 10^3^ CFU/ml in the urine (Food and Drug Administration, 2018).

Clinical failure was defined as the non-resolution of fever (temperature > 38°C) and cUTI symptoms, the development of new symptoms, or all-cause mortality of the patient. Microbiological failure was defined as the pathogen at a colony count exceeding or equalling 10^3^ CFU/ml during or after the treatment. Clinical uncertainty was defined as the loss of urine samples after baseline therapy.

The secondary efficacy endpoint pertained to the determination of sustained microbiological success and all clinical cures. It was measured in terms of the rates of rehospitalization and recurrent cUTIs due to the same ESBL-producing *Enterobacteriaceae* within 3 months of the initial antibiotic treatment.

The safety endpoints included the incidence of *Clostridium difficile* infections, rehospitalization, the loss of intervention and follow-up, and all-cause mortality within 3 months of the first day of effective treatment.

### 2.6 Statistical analysis

The International Business Machines Corporation SPSS Statistics for Windows Version 22.0 and Microsoft Excel (MS Office 2019) were used for data analysis. The analysis population, which included any patients receiving the correct antibiotics, was used to compare the efficacy and safety of the treatment group (PTZ) with that of the control group (meropenem), in terms of the primary and secondary outcomes. The study outcomes were presented using descriptive statistics, such as frequencies and percentages, medians, and interquartile ranges. Medians and interquartile ranges were reported instead of means and standard deviations because the continuous variables were not normally distributed. Nonparametric tests were used in this data analysis. The primary analysis included a chi-squared test to compare the proportion of patients with cUTIs that showed clinical success in the PTZ and meropenem groups. The continuous variables were compared using chi-squared or Fisher’s exact test. For all tests and results, a *p*-value less than 0.05 was considered to denote a statistically significant difference. The strength of association with primary and secondary endpoints was computed using conditional logistic regression adjusted on the study arm. Associations were reported as odds ratios with 95% confidence intervals. Data curation and statistical analyses were performed using Review Manager 5.3.

## 3 Results

### 3.1 Demographic and clinical characteristics of patients

**Figure 1:**
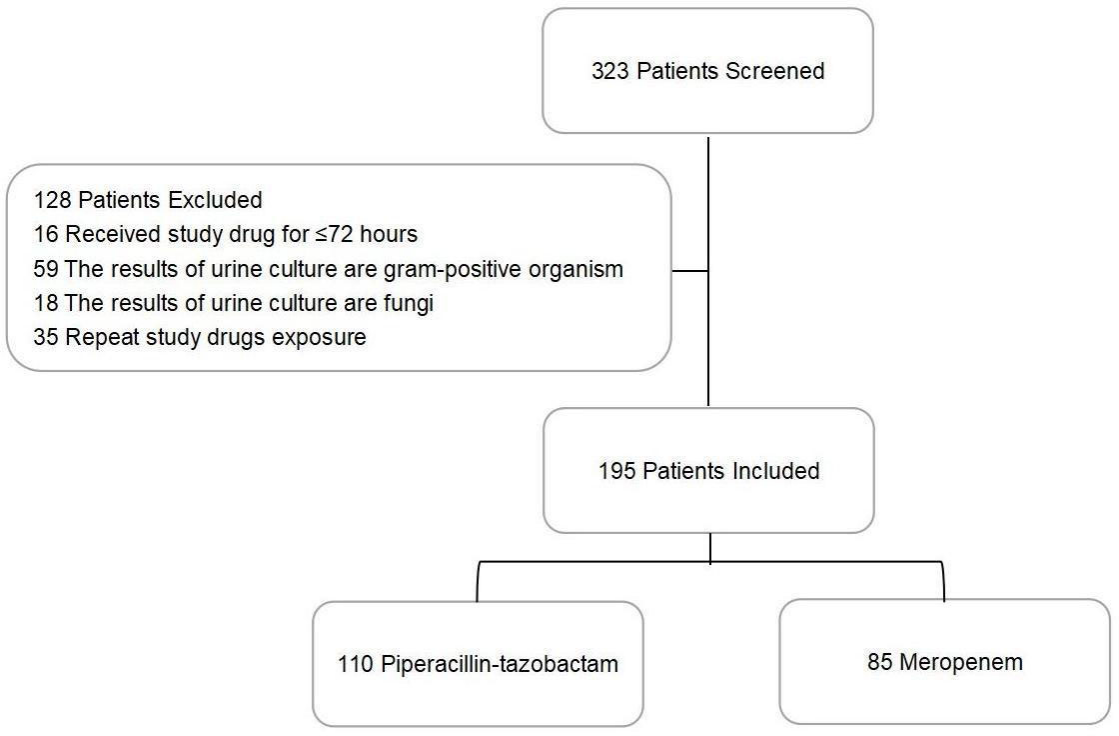
Patient selection procedures.

From a total of 323 patients with cUTIs, 128 were excluded: 16 received antibiotics for less than 72 h, 59 carried Gram-positive organisms in their urine, 18 tested positive for fungi, and 35 had been repeatedly exposed to the studied antibiotics (**Figure 1**). Of the 195 patients eligible for this study, 110 constituted the PTZ group and 85 formed the meropenem group (**Table 1**). Between these two groups, 48 patients (25%) suffered from chronic liver diseases, and 32 (16%) took at least 20 mg of oral corticosteroids daily.

**Table 1:** Patients’ and cUTI characteristics at baseline ^a^.

	PTZ (*n* = 110)	Meropenem (*n* = 85)	Total (*n* = 195)	*p*-value
Patients’ characteristics				
Sex, male (%)	39 (35.5%)	41 (48.2%)	80 (41.0%)	0.072
Age ^b^	65	71	67	**0.033**
Charlson score ^b^,^c^	7	7	7	0.276
Creatinine clearance, eGFR formula ^d^	68 (51–97)	70 (49–98)	68 (50–98)	0.829
SIRS ^e^	55 (50.0%)	46 (54.1%)	101 (51.8%)	0.568
Confounding factors				
Immunocompromised	3 (2.7%)	3 (3.5%)	6 (3%)	0.748
Hematological malignancy	3 (2.7%)	3 (3.5%)	6 (3%)	0.748
Kidney transplant recipient	2 (1.8%)	0	2 (1%)	0.211
Other solid organ transplant recipient	1 (0.9%)	1 (1.2%)	2 (1%)	0.854
Diabetes mellitus	46 (42%)	29 (34%)	75 (38%)	0.273
Chronic kidney disease	41 (37%)	26 (31%)	67 (34%)	0.330
Chronic respiratory disease	8 (7.3%)	9 (11%)	17 (8.7%)	0.416
History of malignancy	20 (18%)	17 (20%)	37 (19%)	0.748
Chronic liver disease	20 (18%)	28 (33%)	48 (25%)	**0.018**
Risk factors for ESBL-producing Enterobacteriaceae				
Chemotherapy within 3 months	11 (10%)	4 (4.7%)	15 (7.7%)	0.169
Daily dose of oral corticosteroids ≥ 20 mg	24 (22%)	8 (9.4%)	32 (16%)	**0.020**
History of UTI	31 (28%)	31 (36%)	62 (32%)	0.218
Recurrent UTI ^f^	25 (23%)	28 (33%)	53 (27%)	0.112
Hospitalization within 3 months	57 (52%)	53 (62%)	110 (56%)	0.141
Invasive procedure within 3 months	19 (17%)	24 (28%)	43 (22%)	0.067
Antibiotics within 6 months	51 (46%)	49 (58%)	100 (51%)	0.118
Fluoroquinolones	19 (17%)	11 (13%)	30 (15%)	0.406
Carbapenems	8 (7.3%)	15 (18%)	23 (12%)	**0.026**
Other β-lactams	24 (22%)	26 (31%)	50 (26%)	0.164
Unknown	15 (14%)	9 (11%)	24 (12%)	0.521
cUTI characteristics				
Signs and diagnosis	85 (77%)	56 (66%)	141 (72%)	0.078
Appropriate antibiotic therapy ^g^	73 (66%)	44 (52%)	117 (60%)	**0.039**
Healthcare associated	57 (52%)	40 (47%)	97 (50%)	0.510
Fever (>38°C)	38 (35%)	44 (52%)	82 (42%)	**0.016**
Chill	8 (7.3%)	12 (14%)	20 (10%)	0.118
Bacteremia	12 (11%)	12 (14%)	24 (12%)	0.499
Required urinary catheterization	33 (30%)	48 (56%)	81 (42%)	**0.000**
Required double-J stent	2 (1.8%)	0	2 (1%)	0.211
ICU admission	11 (10%)	20 (24%)	31 (16%)	**0.010**

ICU, intensive care unit; IQR, interquartile range; SIRS, systemic inflammatory response syndrome.

a Data are expressed as No. (%) of participants unless otherwise indicated.

b Average.

c Charlson comorbidity index (CCI) is a health tool based on the CCI model that assesses the comorbidity risk associated with a series of conditions that offer medical specialists an informed decision-making process, specific screenings, or medical procedures.

d Median.

e SIRS, systemic inflammatory response syndrome.

f Recurrent UTI: Defined as the occurrence of ≥2 UTIs within 6 months, or ≥3 UTIs within a year.

g Appropriateness of antibiotic therapy was defined as an antibiotic therapy active on the strain involved in the current UTI, based on the local procedure for susceptibility testing.

The bold values means there is significant differences in this group of people.

Overall, the baseline characteristics of the two groups were similar (**Table 1**), although the average age and the proportion of patients with chronic liver diseases were both higher in the meropenem group (71 years vs. 65 years, 33% vs. 18%, respectively). In addition, the proportion of patients who received ≥ 20 mg of oral corticosteroids was 22% and 9.4% in the PTZ and meropenem groups, respectively. Before hospitalization and treatment, a larger proportion of patients in the PTZ group were receiving appropriate antibiotic treatments (66% vs. 52%). Moreover, 18% of patients took carbapenem antibiotics in the meropenem group, compared with 7.3% in the PTZ group. More patients from the meropenem group suffered from fever, urethral catheterization, and intensive care unit treatment (52% vs. 35%, 56% vs. 30%, and 24% vs. 10%, respectively). The etiological agents causing cUTIs were similarly distributed between the two groups (**Table 2**): a total of 82 patients (42%) were infected with *E. coli*, 35 (18%) with *K. pneumoniae*, and 11 with *Pseudomonas aeruginosa* (5.6%).

**Table 2:** Microbiological characteristics ^a^.

	PTZ (*n* = 110)	Meropenem (*n* = 85)	Total (*n* = 195)	*p*-value
Gram-negative Enterobacteriaceae				
*Escherichia coli*	60 (55%)	22 (26%)	82 (42%)	**0.000**
*Klebsiella pneumoniae*	20 (18%)	15 (18%)	35 (18%)	0.923
*Enterobacter guspura*	3 (2.7%)	1 (1.2%)	4 (2%)	0.449
*Serratia marcescens*	2 (1.8%)	1 (1.2%)	3 (1.5%)	0.718
*else*	0	1 (1.2%)	1 (0.5%)	0.254
Gram-negative aerobes other than Enterobacteriaceae				
*Pseudomonas aeruginosa*	8 (7.3%)	3 (4.3%)	11 (5.8%)	0.261
*Enterobacter aerogenes*	1 (0.9%)	2 (2.9%)	3 (1.6%)	0.417
*Proteus mirabilis*	2 (1.8%)	1 (1.4%)	3 (1.6%)	0.718
*Citrobacter koseri*	1 (0.9%)	1 (1.4%)	2 (1.1%)	0.854
Drug resistance				
ESBL				
*Escherichia coli*	37 (33.6%)	12 (14.1%)	49 (25.1%)	**0.002**
*Klebsiella pneumoniae*	13 (11.8%)	9 (11%)	22 (11.3%)	0.788
*Enterobacter guspura*	3 (2.7%)	0	3 (1.5%)	-
*Serratia marcescens*	2 (1.8%)	1 (1.2%)	3 (1.5%)	0.718
*Pseudomonas aeruginosa*	4 (3.6%)	1 (1.2%)	5 (2.6%)	0.281
*Enterobacter aerogenes*	1 (0.9%)	2 (2.4%)	3 (1.5%)	0.417
*Proteus mirabilis*	1 (0.9%)	0	1 (0.5%)	-
Amino-R				
*Escherichia coli*	24 (21.8%)	6 (7.1%)	30 (15.4%)	**0.005**
*Klebsiella pneumoniae*	4 (3.6%)	4 (4.7%)	8 (4.1%)	0.709
*Enterobacter aerogenes*	1 (0.9%)	0	1 (0.5%)	-

ESBL, extended-spectrum β-lactamase; Amino-R, aminoglycosides resistant; MIC, minimum inhibitory concentration.

Using MICs from an accompanying antibiotic panel or agar dilution supplemented with glucose 6-phosphate for PTZ/Meropenem, urine isolates were identified to assess patients and microbiologic outcomes. The following definitions were used for this assessment-ESBL: ≥2 µg/ml MIC for aztreonam, ceftazidime, or ceftriaxone; Amino-R: gentamicin ≥8 µg/ml or amikacin ≥32 µg/ml. Patients could have more than 1 isolate from urine sources, and all organisms are presented for completeness. Patients with multiple organisms were counted only once per resistance grouping. If the same species was identified from a different source, the isolate was counted once for the microbiological outcomes.

a Data are expressed as No. (%) of participants unless otherwise indicated.

The bold values means there is significant differences in this group of people.

### 3.2 Microbiology

*E. coli* and *K. pneumoniae* were considered the most common infection-causing pathogens in this study cohort. For the treatment of *E. coli* infections, 55% (60/110) of the patients received PTZ while 26% (22/85) chose meropenem. In contrast, 18% of the patients from both the groups (20/110, 15/85) chose the respective drugs to treat *K. pneumoniae* infections. ESBL-producing and aminoglycosides-resistant (amino-R) *E. coli* were identified as the most resistant pathogens. To combat the former, 33.6% (37/110) and 14.1% (12/85) of the patients used PTZ and meropenem, respectively. For the latter, 21.8% (24/110) and 7.1% (6/110) of the patients chose PTZ and meropenem, respectively. Overall, a higher proportion of patients in the PTZ group tested positive for three different types of *Enterobacteriaceae* in their urine: *E. coli*, ESBL-producing *E. coli*, and amino-R *E. coli* (*p* = 0.000, 0.002, and 0.005, respectively) (**Table 2**).

### 3.3 Treatment

#### 3.3.1 Safety endpoints

The overall duration of antibiotic therapy (9 vs. 6), the effective duration of antibiotic therapy (8 vs. 6), the length of hospital stay (22 vs. 16), and all-cause mortality (18.8% vs. 7.3%) were all higher in the meropenem group than in the PTZ group (**Table 3**). However, no significant difference was observed between the two groups in the relapse of cUTIs, rehospitalization, and loss to follow-up.

**Table 3:** Several occurrences of safety endpoints ^a^.

Endpoints	PTZ (*n* = 110)	Meropenem (*n* = 85)	Total (*n* = 195)	*p*-value
Overall duration of antibiotic therapy ^c^	6 (4–9)	9 (6–14.5)	7 (5–11)	**0.000**
Effective antibiotic therapy duration ^b、c^	6 (4–9)	8 (5–13)	7 (5–10)	**0.000**
Length of hospital stay ^c^	16 (11–30)	22 (15–41)	18 (13–34)	**0.010**
All-cause death	8 (7.3%)	16 (18.8%)	24 (12.3%)	**0.015**
Relapse of UTI	8 (7.3%)	6 (7.1%)	14 (7.2%)	0.954
Re-hospitalization	91 (82.7%)	63 (74.1%)	154 (79%)	0.143
Loss to follow-up	11 (10%)	7 (8.2%)	18 (9.2%)	0.673

a Data are expressed as No. (%) of participants unless otherwise indicated.

b Duration of effective antibiotic treatment: More than 3 consecutive days of therapy was considered as an effective treatment; if not, only continuous time was counted.

c Median.

The bold values means there is significant differences in this group of people.

#### 3.3.2 Efficacy endpoints

**Figure 2:**
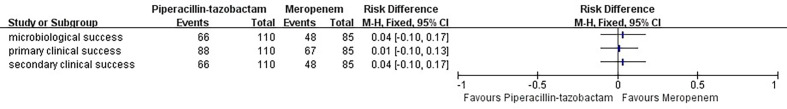
Primary efficacy endpoints.

In terms of clinical cure and microbial eradication, the clinical success of both the groups was comparable. The overall success in the two groups was 80% and 78.8% (treatment difference, 1.2%; 95% confidence interval, 10.3–12.6) (**Table 4**, **Figure 2**).

**Table 4:** Occurrence of primary and secondary endpoints ^a^.

Endpoints	PTZ (*n* = 110)	Meropenem (*n* = 85)	Total (*n* = 195)	*p*-value	Treatment difference (95% CI)
Primary efficacy endpoints					
Success	88 (80%)	67 (78.8%)	155 (79.5%)	0.840	1.2 (10.3–12.6)
Failure	10 (9.1%)	12 (14.1%)	22 (11.3%)		
Indeterminate	12 (10.9%)	6 (7.1%)	18 (9.2%)		
Secondary efficacy endpoint—clinical endpoint response					
Success	66 (60%)	48 (56.5%)	114 (58.5%)	0.620	3.5 (10.4–17.5)
Failure	19 (17.3%)	17 (20%)	36 (18.5%)		
Indeterminate	25 (22.7%)	20 (23.5%)	45 (23.1%)		
Secondary efficacy endpoint—microbiological endpoint response					
Success	66 (60%)	48 (56.5%)	114 (58.5%)	0.620	4.7 (9.3–18.7)
Failure	19 (17.3%)	16 (18.8%)	35 (17.9%)		
Indeterminate	25 (22.7%)	21 (24.7%)	46 (23.6%)		

a Data are expressed as No. (%) of participants unless otherwise indicated.

#### 3.3.3 Microbiological endpoints

Subgroup analyses focused on ESBL-producing and amino-R *E. coli* were conducted. Although clinical cure rates were high and did not differ significantly between the two groups, clinical and microbiological endpoint success was more significant in the PTZ group than in the meropenem group (**Tables 5**–**7**).

**Table 5:** Clinical and microbiologic outcomes among patients with baseline ESBL-producing *Escherichia coli* characteristics (occurrence of primary and secondary endpoints) ^a^.

Endpoints	PTZ (*n* = 37)	Meropenem (*n* = 12)	Total (*n* = 49)	*p*-value
Primary efficacy endpoints				
Success	27 (73%)	9 (75%)	36 (73.5%)	0.890
Failure	3 (8.1%)	1 (8.3%)	4 (8.2%)	
Indeterminate	7 (18.9%)	2 (16.7%)	9 (18.4%)	
Secondary efficacy endpoint—clinical endpoint response				
Success	19 (51.4%)	3 (25%)	22 (44.9%)	0.066
Failure	6 (16.2%)	3 (25%)	9 (18.4%)	
Indeterminate	12 (32.4%)	6 (50%)	18 (36.7%)	
Secondary efficacy endpoint—microbiological endpoint response				
Success	22 (59.5%)	4 (33.3%)	26 (53.1%)	0.115
Failure	6 (16.2%)	1 (8.3%)	7 (12.3%)	
Indeterminate	9 (24.3%)	7 (58.3%)	16 (32.7%)	

a Data are expressed as No. (%) of participants unless otherwise indicated.

**Table 6:** Clinical and microbiologic outcomes among patients with baseline amino-R *Escherichia coli* characteristics (occurrence of primary and secondary endpoints)^a^.

Endpoints	PTZ (*n* = 24)	Meropenem (*n* = 6)	Total (*n* = 30)	*p*-value
Primary efficacy endpoints				
Success	20 (83.3%)	6 (100%)	26 (86.7%)	0.283
Failure	0	0	0	
Indeterminate	4 (16.7%)	0	4 (13.3%)	
Secondary efficacy endpoint—clinical endpoint response				
Success	18 (75%)	3 (50%)	21 (70%)	0.232
Failure	0	2 (33.3%)	2 (6.7%)	
Indeterminate	6 (25%)	1 (16.7%)	7 (23.3%)	
Secondary efficacy endpoint—microbiological endpoint response				
Success	18 (75%)	4 (66.7%)	22 (73.3%)	0.680
Failure	0	1 (16.7%)	1 (3.3%)	
Indeterminate	6 (25%)	1 (16.7%)	7 (23.3%)	

a Data are expressed as No. (%) of participants unless otherwise indicated.

**Table 7:** Clinical and microbiologic outcomes among patients with baseline ESBL-producing and amino-R *Escherichia coli* characteristics (occurrence of primary and secondary endpoints)^a^.

Endpoints	PTZ (*n* = 61)	Meropenem (*n* = 18)	Total (*n* = 79)	*p*-value
Primary efficacy endpoints				
Success	47 (77%)	15 (83.3%)	62 (78.5%)	0.569
Failure	3 (4.9%)	1 (5.6%)	4 (5.1%)	
Indeterminate	11 (18%)	2 (11.1%)	13 (16.5%)	
Secondary efficacy endpoint—clinical endpoint response				
Success	37 (60.7%)	6 (33.3%)	43 (54.4%)	**0.041**
Failure	6 (9.8%)	5 (27.8%)	11 (13.9%)	
Indeterminate	18 (29.5%)	7 (38.9%)	25 (31.6%)	
Secondary efficacy endpoint—microbiological endpoint response				
Success	40 (65.6%)	8 (44.4%)	48 (60.8%)	0.107
Failure	6 (9.8%)	2 (11.1%)	8 (10.1%)	
Indeterminate	15 (24.6%)	8 (44.4%)	23 (29.1%)	

a Data are expressed as No. (%) of participants unless otherwise indicated.

The bold values means there is significant differences in this group of people.

### 3.4 Safety

The incidence of adverse events was significantly lower in the PTZ group (4.5% vs. 15.3%, *p* = 0.01). In the meropenem group, the most common adverse event was abnormal liver function, afflicting 5.9% of the patients. In the PTZ group, the most common adverse event was anaphylaxis, affecting 2.7% of the patients (**Table 8**).

**Table 8:** Adverse patient events ^a^.

	PTZ (*n* = 110)	Meropenem (*n* = 85)	Total (*n* = 195)	*p*-value
No. of all adverse events (%)	5 (4.5%)	13 (15.3%)	1 (0.5%)	**0.01**
Diarrhea	0	1 (1.2%)	1 (0.5%)	–
No. with thrombocytosis ^b^ (%)	0	4 (4.7%)	4 (2.1%)	–
No. with thrombocytopenia ^c^ (%)	2 (1.8%)	1 (1.2%)	3 (1.5%)	0.718
No. with bone marrow suppression ^d^(%)	0	1 (1.2%)	1 (0.5%)	–
No. with hepatic insufficiency (%)	0	5 (5.9%)	5 (2.6%)	–
No. with rash (%)	3 (2.7%)	1 (1.2%)	4 (2.1%)	0.449

a Data are expressed as No. (%) of participants unless otherwise indicated.

b Thrombocytosis = blood cell count of ≥450 cells × 10^9^/L.

c Thrombocytopenia = blood cell count of <150 cells × 10^9^/L.

d Bone marrow suppression = with leukopenia count (3.0–3.9) × 10^9^/L, hemoglobin count 95–100 g/L, blood cell count (75–99) × 10^9^/L.

The bold values means there is significant differences in this group of people.

## 4 Discussion

According to the FDA (US Food & Drug Administration, 2018) and the Association of the British Pharmaceutical Industry, real-world evidence is defined as the analysis of real-world clinical data to evaluate what is happening in normal clinical practice. Following appeals from many agencies, studies focusing on real-world evidence have received much attention. Different from randomized clinical trials, clinical studies in the real-world setting deal with larger populations with complex situations, physical functions, and variable treatment combinations. Such studies represent the general population, provide deeper insights into clinical practice, and help discover real-life problems (Kim et al., 2018).

However, clinical retrospective studies in the real world suffer from some unavoidable drawbacks. The healthcare practitioners directly decide the drugs to be administered after analyzing multi-indicators. Changing the medicines for finishing the clinical study without considering clinical ethics is illegal.

This study aimed to compare the efficacy and safety of PTZ and meropenem in treating cUTIs in the real world. In this monocentric and case–control study, patients diagnosed with cUTIs received antibiotic therapy, and the clinical cure rate was high (79.5%) at the end of the treatment. The clinical characteristics of patients who took PTZ and meropenem were similar, and their clinical response rates were 80% and 78.8%, respectively. Furthermore, the clinical response did not differ significantly among patients with several health conditions, including immunocompromised patients and those with hematological malignancies, kidney and other solid organ transplantations, diabetes, chronic kidney disease, chronic respiratory disease, and other malignancies.

The higher clinical response of PTZ (80%) was not related to the microbiological or clinical characteristics of the patients. PTZ was used at a standard dosage of 4.5 g q8h, or adjusted according to creatinine clearance for pathogenic infections with a minimum inhibitory concentration ≤ 8 mg/L. In this study, the most common pathogens were *E. coli* (42%) and *K. pneumoniae* (18%), and one-third of the patients were diagnosed with diabetes mellitus or chronic kidney diseases.

The clinical efficacy and safety of PTZ and meropenem were similar in treating cUTIs. However, the increased use of carbapenems may lead to the selection of carbapenem resistance in Gram-negative bacilli and the spread of carbapenemase-encoding antibiotic resistance genes (Mendes et al., 2018). To avoid serious outcomes and achieve appropriate prescriptions, many studies have compared carbapenems with other antibacterials (Chen et al., 2018; Sternbach et al., 2018; Zhong et al., 2018; Ezure et al., 2022). In these meta-analyses, carbapenems present similar clinical efficacies and possibly better microbiological responses than other antibacterial agents. When using antibiotics, the possibility and consequences of resistance and related health economics must be considered.

The efficacy of drugs varies with the patients’ physical conditions, complications, and history of taking other drugs. We, as clinical practitioners, found that there is a lack of therapeutic advice on drugs that share similar indications or targeting the same pathogens, to help physicians deliver more effective, precise, and cost-effective treatments to different patients. More clinical real-world data need to be collected and analyzed to construct detailed guidelines for drug administration. These studies should be designed based on the standard format of medical records between multiple centers and should overcome limitations as much as possible.

## 5 Limitations

This study had several limitations. Firstly, this monocenter and retrospective study was limited by an insufficient sample size and a small number of case events, which led to restricted statistical power to manage confounding factors. Secondly, the extensive use of PTZ in the first line resulted in a population selection and biased measurement of point estimates. Only randomization can prevent such biases. Lastly, other antibiotics in clinical practice may influence the clinical efficacy and incidence of adverse effects.

## 6 Conclusions

PTZ is an effective, safe, and definite treatment option for cUTIs due to the presence of ESBL-producing and amino-R *Enterobacteriaceae*. Its efficacy is consistent with that of immunocompromised patients, including those with hematological malignancies, kidney and other solid organ transplant recipients, diabetes mellitus, chronic kidney disease, chronic respiratory disease, and a history of malignancies. However, the risk of emerging resistance stresses the need for close monitoring. A randomized comparison with carbapenems is warranted.

## Data availability statement

The data analyzed in this study is subject to the following licenses/restrictions: Hospital Personal Information. Requests to access these datasets should be directed to 1358359@qq.com.

## Ethics statement

The studies involving human participants were reviewed and approved by the Medical Ethics Committee of the First Affiliated Hospital of the Air Force Medical University. The patients/participants provided written informed consent to participate in the study. Written informed consent was obtained from the individual(s) for the publication of any potentially identifiable images or data included in this article.

## Author contributions

J-WW designed the study and approved the final version of the manuscript. WZ, C-YY, T-TF, YG, and S-RL performed the study. WZ and C-YY analyzed the data and wrote the manuscript. S-SC, BC, M-YL, B-YF, BJ, LiW, FC, JC, and LeW were involved in conceptualization, methodology, and analysis of the study. BC and T-TF provided statistical analysis assistance and helped to revise the manuscript. All authors have read and approved the final manuscript.
